# Hepatitis B vaccination coverage among healthcare workers at a tertiary hospital in Rwanda

**DOI:** 10.1186/s13104-018-4002-5

**Published:** 2018-12-13

**Authors:** Claude Mambo Muvunyi, Jean De Dieu Harelimana, Osee Rurambya Sebatunzi, Aschariya Chinma Atmaprakash, Eric Seruyange, Florence Masaisa, Olivier Manzi, Martin Nyundo, Théobald Hategekimana

**Affiliations:** 1 Department of Clinical Biology, School of Medicine and Pharmacy, College of Medicine and Health Sciences, University of Rwanda, Kigali, Rwanda; 2 Department of Biomedical Laboratory Science, School of Health Science, College of Medicine and Health Sciences, University of Rwanda, Kigali, Rwanda; 3 Department of Internal Medicine, School of Medicine and Pharmacy, College of Medicine and Health Sciences, University of Rwanda, Kigali, Rwanda; 4 Kigali University Teaching Hospital, Kigali, Rwanda

**Keywords:** Health care workers, Hepatitis B, Vaccination, Immune response

## Abstract

**Objective:**

We evaluated post-vaccination immunity status and describe potential risk factors associated with the lack of response among healthcare workers (HCWs) at a tertiary care hospital in Kigali, Rwanda.

**Results:**

Of 373 HCWs, 291 (78.2%) were female and 81 (21.8%) were male. The mean age of the study participants was 40.2 years (standard deviation [SD], 7.7 years), within a range of 24–41 years. Participants’ mean BMI was 25.4 ± 6.6 kg/m^2^, with more than half of patients (60.3%) being overweight. 96% received all three doses of vaccination. A total of 36 participants (9.6%) were considered non responders as they did not develop a sufficient anti-HBs response post vaccination. The anti-HBs response was significantly higher in females when compared to males (p = 0.02). Interestingly, there was no significant association between decline in antibody levels with age (*p* = 0.242) and BMI (*p* = 0.516) of the participants. The anti-HBs titers were similar in the group of participants who had received two doses and those who had received three doses of the HBV vaccination. Overall the findings of our study provide a basis for testing for anti-HBs in all HCWs post vaccination in Rwanda.

## Introduction

Hepatitis B virus (HBV) infection is a major public health problem associated with high morbidity and mortality associated with many long-term complications including chronic liver disease, cirrhosis and hepatocellular carcinoma. Approximately 350 million patients around the world are chronically infected with HBV and about 500,000 patients die every year from end stage complications of persistent infection. Those with chronic infection constitute the primary reservoir of HBV infection that can be transmitted through blood and body fluids [1]. Studies conducted in Taiwan and India suggest that a non-vaccinated individual faces a risk ranging from 6 to 30% of acquiring HBV infection after only a single exposure to HBV infected blood or body fluids [2, 3]. Although there has been much progress on antiviral therapy, only a minority of chronic hepatitis B patients have a sustained cure response. Thus primary prevention by vaccination remains the only effective protective measure in reducing the incidence of HBV infection and chronic liver disease and diminishing the pool of chronic carriers, thus limiting transmission of infection to non-vaccinated contacts. Health care workers (HCWs) are at high risk of HBV infection due to frequent occupational exposure to blood and potentially infectious body fluids [4]. Previous reports have shown up to a fourfold increase in the risk of acquiring HBV infection in HCWs as compared to the general population. Thus, some countries have established HBV vaccination policies for HCWs [5, 6]. In Rwanda, researchers revealed a prevalence of 2.9% of acute and chronic HBV infection among medical staff at tertiary and teaching hospitals [5]. In response to those findings, the Rwanda Ministry of Health issued a recommendation to vaccinate all HCWs and medical students who were born after the year 2002 when hepatitis B vaccine was introduced in the expanded program of immunization (EPI). From 2013 HBV vaccines were administered to all medical staff and also provided to any new employee in Rwandan healthcare facilities. The HBV vaccination is scheduled in three intramuscularly injections following recommendations from the EPI. Generally, there is no need for booster doses for fully vaccinated immunocompetent individuals but a proportion of individuals do not respond to the standard three doses of HBV vaccine [7]. Therefore, individuals at higher risk, such as HCWs, represent a group in which post vaccination evaluation and regular monitoring of antibodies development is recommended [8]. As recommended by World Health Organization (WHO), post-vaccination antibody level is generally measured 6–8 weeks following completion of 3 vaccine doses to detect the immune response. The measured level of anti-HBs ≥ 10 mIU/mL is generally considered as protective. Several studies in Rwanda reported an absence or lowered response rates in 12%–22% HCWs in response to HB vaccinations [9, 10]. However, in Rwanda, there are no strict post-vaccination policies to confirm and monitor the development of immunity from the vaccine of HBV, nor have any assessments of post-vaccination seroconversion among high risk groups, especially HCWs been carried out. Results from antibody surveillance could allow the identification of medical staff at risk of acquiring HBV and in need of revaccination. This study aimed to evaluate post-vaccination immunity status and characterize potential risk factors that may be associated with the lack of response among HCWs.

## Main text

### Materials and methods

#### Study design and settings

A collaboration team including members from the Infection and Prevention and Control Committee and laboratory staff conducted an analytical and observational cross-sectional study at Centre Hopstalo-Universitaire de Kigali (CHUK) with HCWs who have completed the hepatitis B vaccination schedule. Participants included HCWs who had received the three compulsory doses of HBV vaccine series between 2013 and 2015.

#### Sample collection and sampling strategies

After written informed consent, a structured questionnaire was administered to all participants to obtain demographic information, socioeconomic status and other relevant information on risk factors for low antibody level of HBV vaccine. We collected 3–4 mL of blood specimens from HCWs at CHUK. Serological assay was carried out to assess the level of antibodies in response to hepatitis B vaccine. The samples were analyzed in CHUK’s microbiology department. We performed serological testing for HBV status using commercially available enzyme linked immunosorbent assays (ELISA) on serum samples prepared from venous blood, for anti-HBs to assess the level of protection. HCWs with non-sero-protective levels of anti-HBs titre (< 10 IU/L) were to be revaccinated with a three doses series. Documentation of primary HB vaccination series record was confirmed by checking the date and dose intervals of HB vaccine in their personnel files.

The target study enrolment consisted of HCWs randomly selected and who were vaccinated from 2013, the year in which the hepatitis B vaccine was introduced into these settings. The participants were adults aged 18 years and above who had completed the course of vaccination with doses of ENGERIX B (1 mL each) administered intramuscularly and given on an 1-, 3- and 6-month schedule. We collected a total of 373 serum samples from HCWs who were vaccinated during the period of 2013 until 2016. In this study, antibody levels below protective level (10 mIU/mL) were considered non-responders. CHUK Ethics and Research Committee approved the study.

#### Statistical analysis

We performed all statistical analyses using the Statistical Package for the Social Sciences (SPSS), version 17.0 for Windows (SPSS, Inc. Chicago, IL). First, we used descriptive statistics, including count and percentage, to describe the demographic characteristics of the participants. We computed the mean and standard deviation for quantitative data variables while we compared qualitative data using proportions. We performed univariate analysis for associations between identified potential risk factors and their potential association with lack of response among HCWs using Chi square tests for discrete variables, with p < 0.05 considered statistically significant.

### Result

#### Socio demographic characteristics of HCWs

Socio demographic characteristics of the participants enrolled in this study are described in Table 1. 291 (78.2%) participants were female and 81 (21.8%) were male. Out of 369 participants with complete information on age, 69.4% were in the age group of 30–44 years, 23% were over 45 years, and 7.6% below 30 years. The mean age of the study participants was 40.2 years (standard deviation [SD], 7.7 years), within a range of 24–41 years. Participants’ mean BMI was 25.4 ± 6.6 kg/m^2^, with more than half of patients (60.3%) considered overweight. 18.9% of participants reported alcohol consumption while only two participants were active smokers. Over half the participants (56.8%) were nurses and 23.9% of participants were from the department of internal medicine.

**Table 1:** Demographic characteristic of study participants

	Frequency	Percentage (%)
Age, years (n = 369)		
Age mean (SD) = 40.2 (7.7) Age median (IQR) = 39 (41)		
< 30	28	7.6
30–44	256	69.4
≥ 45	85	23
Gender (n = 372)		
Male	81	21.8
Female	291	78.2
BMI, kg/m^2^ (n = 310)		
BMI, kg/m^2^ mean (SD) = 25.4 (6.6)		
< 25	123	39.7
25 to < 30	120	38.7
≥ 30	67	21.6
Alcohol (n = 369)		
Yes	44	18.9
No	325	88.1
Smoking (n = 371)		
Yes	2	0.6
No	369	99.4
Occupation (n = 373)		
Physician	10	2.7
Resident	34	9.1
Nurse	212	56.8
Midwife	43	11.5
Laboratory technician	19	5.1
Other^a^	55	14.8
Department of work (n = 373)		
Emergency	34	9.1
Internal medicine	89	23.9
Surgery	54	14.5
Gynecology	53	14.2
Pediatrics	40	10.7
Pathology	24	6.4
Other^a^	79	21.2
Number of doses (n = 373)		
One dose	0	0
Two dose	15	4
All three dose	358	96

^a^ICU/anesthesiology, pharmacy, physiotherapy, radiology, ophthalmology

#### Immune response to HBV vaccine and its relation to some socio demographic factors

Participants’ adherence to the vaccination schedule and the immune response to HBV vaccine as measured by anti-HBs titers are illustrated in Tables 1 and 2 respectively. Of the total participants, 358 (96%) had received all three doses of vaccination and the rest (4%) had received two doses only. Overall, the majority of those vaccinated (70%) had a high level of immune response (i.e. anti-HBs > 100 mIU/mL) 20.4% were hyporesponders (i.e. anti-HBs titer between 10 and 100 mIU/mL) and 9.6% were classified as nonresponders because they did not develop a sufficient anti-HBs response (Table 2).

**Table 2:** Distribution of responder type based on anti-HBs titres

Type of response (n = 373)^a^	Frequency	Percentage (%)
Non-responsive	36	9.6
Hypo-responsive	76	20.4
Hyper-responsive	261	70

^a^Non-responsive, < 10 mIU/mL); hypo-responsive, between 10 and 100 mIU/mL; hyper-responsive, ≥ 100 m IU/mL

#### Risk factors for non-response to HBV vaccine

Bivariate analysis of risk factors for non-response to HBV vaccine is described in Table 3. The anti-HBs response was significantly higher in females when compared to males (p = 0.02). There were no significant associations between decline in antibody levels with age (*p* = 0.242) and BMI (*p* = 0.516) of the subjects. The anti-HBs titers were similar in the group of participants who had received two doses and those who had received three doses of the HBV vaccination.

**Table 3:** Bivariate analysis of risk factors for non-response to HBV vaccine

Variables	Non-responsive, N (%)	Hypo/hyper-responsive, N (%)	OR (95% CI)	*p* value
Age, years				
< 40	18 (9.6)	169 (90.4)	1	
≥ 40	18 (9.9)	164 (90.1)	1 (0.5–1.9)	0.932
Gender				
Female	23 (7.9)	268 (92.1)	1	
Male	13 (16)	68 (84)	1.8 (1.1–2.9)	0.02
BMI, kg/m^2^				
< 25	13 (10.6)	110 (89.4)	1	
≥ 25	20 (9.7)	186 (90.3)	1.1 (0.5–2.3)	0.802
Number of dose				
2 doses	1 (2.9)	33 (97.1)		
All three dose	5 (1.6)	316 (98.4)	1 (0.9–1.1)	0.5

### Discussion

The Government of Rwanda implemented an immunization plan for HCWs and medical students in 2013 to protect them from occupational exposures and transmission. However, a proportion of individuals do not respond to the recommended standard three dose HBV vaccination and remain susceptible to the infection. This is the first study from Rwanda that provides local epidemiological data assessing the immune response and predictors of non-response to HBV vaccine in HCWs since its implementation. In our study, 9.6% of HCW did not develop a protective level (< 10 mIU/mL) of anti- HBs, a proportion which is comparable to the global level for a poor immune response to HBV immunization of 5–10% [10]. Our findings that about 9.6% of the population were non-responders is also in agreement with a report by Chathuranga et al. in Sri Lanka where 9.9% of HCWs did not develop a protective level of anti-HBs [11].

The majority of our sample were in the age range of 30–44 years with a mean age of 40.2 years which is similar to study by Chathuranga et al. but differ from that of Sahana et al. where majority were in the age range of 18–24 years [12]. 78.2% of HCWs were females which is similar to study by Rao et al. whereas two other studies showed male predominance [13–15]. Of the total participants, 96% had received all three doses of vaccination and the rest (4%) had received two doses only. The results from our study was found to be higher when compared to other studies done elsewhere [16–18]. This high adherence to the dosing schedule is likely due to the policy issued by the MOH in 2013 to vaccinate all HCWs.

Another important objective in this study was to analyze gender, age, BMI and vaccine status in the study sample in relation to the pattern of HBV vaccine response. The percentage of male non-responders (16%) was higher than female (7.9%) and similar findings have been reported in previous studies [11, 19]. Smoking and alcohol have been proposed as probable reasons for a poor immune response to HBV immunization in men; however, these were not evaluated in our study due to the low proportion of non-responders to the vaccine found in the present study. Our literature review has revealed that seroconversion to anti-HBs is higher when age at vaccination is > 40 years compared to when age at vaccination is above 40 years [19, 20]. However, our results did not show an association between age at vaccination and the rate of seroconversion to anti-HBs; perhaps because the majority of our study population was younger than 40 years of age. Similarly, BMI in our study was not significantly different between vaccine responders and non-responders as observed in a study by Hussein et al. in Egypt. Furthermore, in our study, the anti-HBs titers were similar in the groups of participants who had received two and three doses and of HBV vaccination. This is of particular importance and reassurance for protection in settings that have reported low HBV vaccination rate in HCWs.

In conclusion, the immune response after completion of scheduled of a standard HBsAg immunization in HCWs (90.4%) was similar to that observed in HCWs in other parts of the world, with gender being the only factor associated with poor response. Although protection was obtained in HCWs who received three and two HBV vaccine doses, the findings of our study provide a basis for testing for anti-HBs in all HCWs in Rwanda 6–8 weeks post vaccination. Confirmatory testing of immune response will not only ensure safety of HCWs but also reduce rate of transmission resulting in a cost-effective strategy for individuals as well as at the national level.

## Limitations

Although the study successfully demonstrated that immune response after HBV vaccination is similar to that observed in other parts of the world, it has certain limitations in terms of predictors of non-response to the vaccine. In the current study, we were unable to analyze variables such as genetic factors know to impact the response to vaccination.

## Abbreviations

BMI: Body Mass Index
CHUK: Centre Hospitalo-Universitaire de Kigali
ELISA: Enzyme-Linked Immunosorbent Assay
EPI: Expended Program of Immunization
HBV: hepatitis B virus
HCW: healthcare worker
IQR: interquartile range
SD: standard deviation
SPSS: Statistical Package for the Social Sciences
WHO: World Health Organization
